# Impact of SARS-CoV-2 Infection on the Epidemiology of Chronic Pain and Long-Term Disability: Prepare for the Next Perfect Storm

**DOI:** 10.3389/fpain.2020.616284

**Published:** 2020-12-15

**Authors:** Guy Henri Hans, Davina Wildemeersch

**Affiliations:** ^1^Antwerp University Hospital, Antwerp, Belgium; ^2^University of Antwerp, Antwerp, Belgium

**Keywords:** COVID-19, neuropathic pain (NP), chronic pain, hyperalgesia, cytokine—immunological terms, vitamin D

Chronic pain is the leading cause of disability in the world. Its prevalence has increased worldwide to affect more than 15% of the world population. Access to pain treatment has been deemed a fundamental human right by the World Health Organization. The current severe acute respiratory syndrome coronavirus 2 (SARS-CoV-2) pandemic could lead to a significant worsening of the epidemiology of chronic pain. Several pathophysiological, environmental, and psycho-social causes can be expected to result in a worldwide surge in chronic pain complaints.

From a pathophysiological standpoint, it has become clear that the SARS-CoV-2 infection is characterized by the occurrence of a cytokine storm resembling that leading to macrophage activation as seen in virus-induced hemophagocytic lymphohistiocytosis. The cytokine profile of coronavirus disease (COVID-19) is associated with its severity and is characterized by increased levels of interleukin (IL)-2, IL-7, and other cytokines, such as IL-6. The prospect of an autoimmune component in the pathogenesis of SARS-CoV-2 infection is however challenging from a nociceptive standpoint. Cytokines have indeed previously been recognized as being important in the development of allodynia and hyperalgesia in diverse pathological pain conditions. This is probably due to an inflammatory mediator-induced hyperalgesia, which is sometimes referred to as hyperalgesic priming (1). SARS-CoV-2 infection could lead to a state of hyperstimulation resulting in autoimmunity. This auto-immune state can in turn lead to mitochondrial dysfunction, resulting in a state of pronounced neuro-inflammation. Vitamin D deficiency, which has recently been observed in a large proportion of COVID-19 patients with need for ICU admission, will further enhance this neuro-inflammatory condition (2). The pronounced neuro-inflammatory condition can result in the development of small fiber neuropathy (SFN), leading to pain and decreased production of anti-inflammatory peptides. This can lead to a further enhancement of the hyperstimulated state. In addition, many patients recovering from the SARS-CoV-2 infection display signs of critical illness polyneuropathy. There are thus several reasons why COVID-19 patients are prone to the development of painful polyneuropathies, which can negatively impact their quality of life.

Furthermore, COVID-19 patients frequently show neurological manifestations directly caused by the SARS-CoV-2 infection. In past outbreaks of SARS-CoV, it has been shown that the virus targets the central nervous system (CNS) leading to distinct signs of neuro-inflammation. Such post-inflammatory CNS pathology can adversely affect sleep, pain sensitivity, and energy. Furthermore, prolonged administration of opioids in the intensive care unit can induce a state of enhanced pain sensitization in patients, leading to symptoms of allodynia and hyperalgesia along with myalgia, cramping, and even abdominal pain. Such a condition is often referred to as opioid-induced hyperalgesia (OIH) and has been recently considered as a neurotoxic condition (1). Dynorphin may be involved in the development of OIH. Dynorphin is an endogenous opioid peptide that binds primarily to the kappa opioid receptor and NMDA receptors, albeit to a lesser extent. It is believed that dynorphin's agonism of the kappa opioid receptor and NMDA receptor has a role in the pronociceptive effect in OIH. Dynorphin also causes the release of excitatory neuropeptides within different locations in the CNS, further enhancing the development of a central neurotoxic state.

Such clinical syndromes, although mainly observed in adults, can also be noted in children and adolescents as painful COVID-19-related syndromes. In North America and Europe, at least 800 children have been diagnosed with a strange syndrome that is likely to be associated with COVID-19. The illness appears to be similar to toxic shock syndrome and Kawasaki disease, a rare but serious childhood heart condition; however, its manifestations do not perfectly correspond to those of either shock syndrome or Kawasaki disease. Experts refer to it as multisystem inflammatory syndrome in children (MIS-C). Affected children develop extreme pain throughout the entire body in the acute phase of the disease. Currently it is not yet known why a small fraction of children develop MIS-C while the overwhelming majority of children with COVID-19 recover without problems. A small sample study from France indicated that treatment with immunoglobulin and steroids could yield positive outcomes in children with COVID-related MIS-C (3).

It has been previously shown that survivors of severe acute respiratory syndrome can develop a type of post-SARS sickness syndrome, a disease entity that is characterized by persistent fatigue, weakness, and severely limited exercise tolerance (4). The features of such post-SARS syndrome overlap with the clinical and sleep-related features of widespread pain and chronic fatigue syndromes. This complex of symptoms could be the result of the underlying SFN, a chronic post-inflammatory CNS-related pathological condition and a state of dysautonomia that could be the result of the initiation of an autoimmune reaction. An increasing number of observations in COVID-19 patients requiring hospitalization indicate that these patients indeed suffer from prolonged general fatigue, orthostatic intolerance, regional pain syndromes, and cognitive impairment. Long-lasting cognitive complaints are associated with anxiety and depression but could also negatively impact patient compliance to anti-nociceptive therapies (5).

The 2019 Corona Virus Disease causes universal psychosocial impact, impacting multiple sections of society including children, adults but also elderly. Apart from physical suffering, the consequences of imposed mass quarantine and nationwide lockdown programs on the mental health and well-being at personal and population-levels are many fold, leading to pronounced anxiety, loneliness and distress (6). The strongest association between loneliness related to isolation and mental illness is depression (7). In addition, a very recent study indicated that survivors of COVID-19 appear to be at increased risk of psychiatric sequelae (8). It is therefore to be expected that during and after the pandemic anxiety and depression will increase at a population level. Pain and depression display a strong mutual correlation whereby distress and depression predispose to chronic pain and, inversely, chronic pain predisposes to depressive-like behavior. Chronic pain and depression may be based on common neuroplasticity mechanism changes, which are a potentially important route for the onset and worsening of chronic pain and depression (9). In particular, injury sensory pathways of body pains have been shown to share the same brain regions involved in mood management, forming a histological structural foundation for the coexistence of pain and depression. In addition, animal studies have shown that social defeat stress induces hyperalgesia regardless depressive-like behavior in the animals (10).

Special attention needs to be attributed to the development of long-lasting and disabling headache after acute infection with SARS-CoV-2 (11). During the acute phase the headaches have migraine-like features, including a throbbing or pressing nature. Aggravation with bending over is often reported, as well as sensory disturbances such as photophobia and phonophobia. Pathophysiologically, its migraine-like features may reflect an activation of the trigeminovascular system by inflammation or direct involvement of SARS-CoV-2. It has been hypothesized that the virus has neurotropic characteristics, being able to invade peripheral nerve terminals but also enter the central nervous system through trans-synaptic pathways. Inside the nasal cavity, concomitant anosmia may indicate a peripheral activation of the trigeminovascular system. Some reports indicate that headache may be more common in patients with gastrointestinal symptoms. One study suggested that rapid weight loss with reduced appetite are distinctive features of COVID-19 headache (12). In the chronic phase the headaches seem to evolve into a more constant nature with significant negative impact on health-related quality of life in these patients.

The challenge of reintegrating survivors of COVID-19 into the workplace will be vital for the recovery of the economy. These patients will however not only require prolonged rehabilitation but are also in need of integrated multidisciplinary anti-nociceptive therapeutic approaches. Some of the symptoms presented by such survivors should be considered as psychological traumatic effects of the acute infectious illness. These symptoms may also be the result of their prolonged isolation from family and friends, while being faced with an uncertain outcome and the threat of death. Globally, the World Health Organization estimates that 30–50% of the population affected by a disaster suffer from diverse psychological distress. Countries should therefore monitor survivors of COVID-19 to identify risk factors for the development of post-traumatic stress syndrome (PTSD) and chronic pain and primary pain (widespread pain) syndromes. PTSD is commonly comorbid with chronic pain conditions. Patients with pain and PTSD as a comorbidity report significantly greater pain severity and have a higher number of complaints of pain. This may be attributable to the hyperalgesic state often observed in PTSD patients, which may in turn be related to central sensitization. Furthermore, 39.7% of patients with neuropathic pain conditions have PTSD, which is much higher than the prevalence of PTSD in the general population with chronic pain (9.8%) (13).

From a broader societal perspective, it should be noted that even the management of pain syndromes in non-COVID-19 patients has been greatly affected by the current COVID-19 pandemic. Elective pain management procedures have been postponed, sometimes abruptly interrupting long-term planned approaches to manage chronic pain conditions. However, even after the procedures are performed, some patients are reluctant to resume their medical follow-up schedule. Further, patients are reluctant to go to hospitals or care facilities, further delaying necessary care. The long-term effects of the pandemic will not be known until much later and likely include disease flare-ups owing to stressors and discontinuation of pain medications, PTSD, and decreased number of physician visits with subsequent under-treatment and further deterioration of chronic pain conditions. Adequate surveillance and monitoring systems should be implemented as from now, to detect the suspected rise in prevalence of pain as a result of this pandemic. Such monitoring systems should be not only tailored to adults but also to children and adolescents as they can also suffer from long-term disability after the current pandemic.

Altogether, survivors of COVID-19 are prone to the development of chronic (neuropathic) pain states. The epidemiology of PTSD, chronic pain, persistent sleep disorders, fibromyalgia, severe depression and fatigue in COVID-19 survivors will need to be extensively studied. Further, even non-COVID-19 patients with pre-existing chronic pain conditions, an already vulnerable patient population, are prone to severe consequences of the current pandemic. After the acute infectious storm subsides, a nociception- and disability-related tsunami is expected to occur, presenting an entirely novel set of challenges which the international healthcare community needs to be prepared for.

## Author Contributions

GH was responsible for the content of the manuscript and the first draft. DW further developed the content of the manuscript and finalized the paper. All authors contributed to the article and approved the submitted version.

## Conflict of Interest

The authors declare that the research was conducted in the absence of any commercial or financial relationships that could be construed as a potential conflict of interest.
